# Surgical Management of Massive Pulmonary Embolism Presenting with Cardiopulmonary Arrest: How Far Is Too Far?

**DOI:** 10.21470/1678-9741-2021-0354

**Published:** 2023

**Authors:** Kaushalendra Rathore, Mark Newman

**Affiliations:** 1 Department of Cardiothoracic Surgery, Sir Charles Gairdner Hospital, Nedlands, Australia.

**Keywords:** Thrombosis, Pulmonary Embolism, Shock, Heart Arrest, Embolectomy

## Abstract

The incidence of diagnosed massive pulmonary embolism presenting to the Emergency Department is between 3% and 4.5% and it is associated with high mortality if not intervened timely. Cardiopulmonary arrest in this subset of patients carries a very poor prognosis, and various treating pathways have been applied with modest rate of success. Systemic thrombolysis is an established first line of treatment, but surgeons are often involved in the decision-making because of the improving surgical pulmonary embolectomy outcomes.

## INTRODUCTION

Approximately 20% of the patients presenting with pulmonary artery embolus can have massive pulmonary embolism (MPE), and it has a very high mortality rate in the first hour of presentation if it is not intervened promptly^[1]^. Treating teams can be perplexed about the best possible management strategy in crunch time situations of haemodynamic instability progressing to cardiac arrest. Improving surgical outcomes of the index surgery as well as need for mechanical support devices in these unstable patients bring cardiac surgeons in the middle of decision making^[2,3]^. There are numerous unanswered questions in this field when patient is referred with ongoing cardiopulmonary resuscitation (CPR), and there is still no consensus about the standard pathway to route these patients^[4]^. Here we present the surgeon’s perspective on this subject with the background of our own prior and continuing experience in this field^[5,6]^.

## QUESTIONS

**A.** Do surgeons have any role in the management of MPE?
**B.** If at all, then when to intervene and how to manage these sick cases?
**C.** Role of venoarterial extracorporeal membrane oxygenation (VA-ECMO).
**D.** Surgical pulmonary embolectomy (SPE) *vs.* catheter-based thrombolysis.
**E.** How far is too far? Where to stop?

## Discussion of Questions

**Fig. 1:**
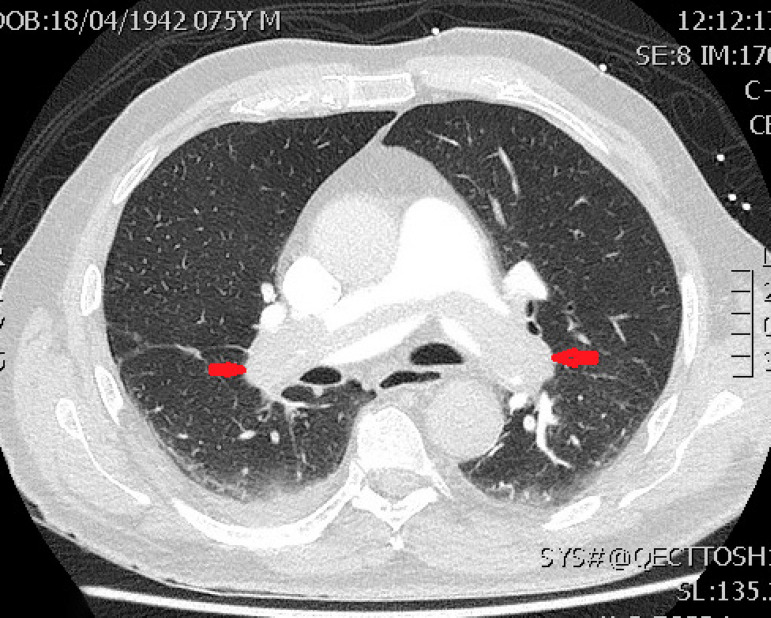
Computed tomography of pulmonary artery (axial view). Arrows show saddle pulmonary embolus obliterating bilateral pulmonary inflow.

**Fig. 2:**
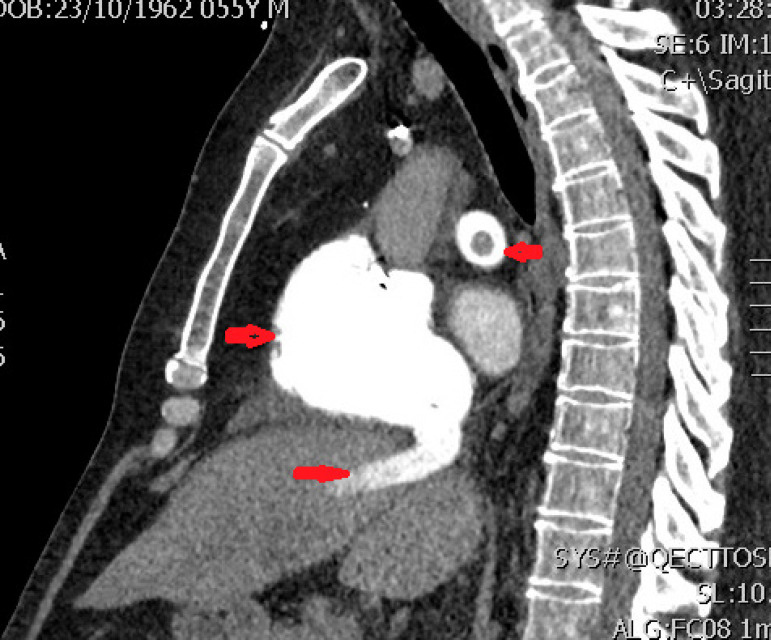
Computed tomography of pulmonary artery (sagittal view). Arrows show right ventricular dilatation with reverse flow in inferior vena cava and clot in the pulmonary artery.

**Fig. 3:**
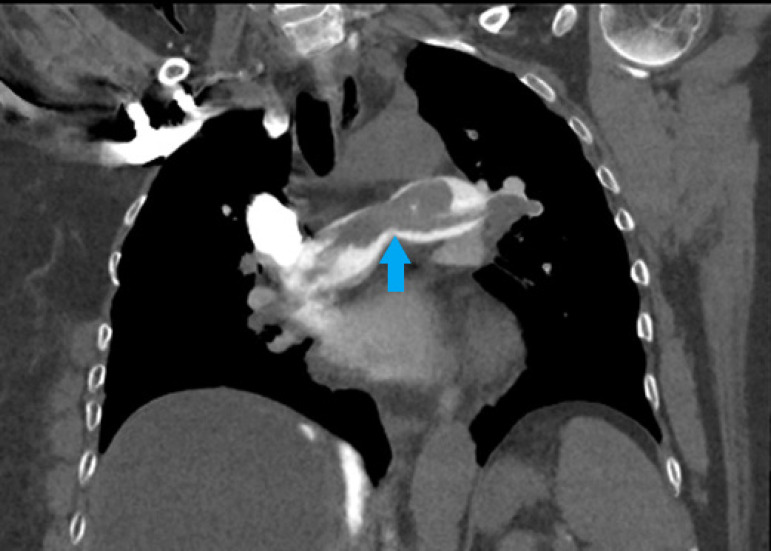
Computed tomography of pulmonary artery (coronal view). Arrow shows extensive clot burden in the pulmonary arteries with potential for distal thromboembolism.

**A.** Clots in the pulmonary arteries do not allow proper forward flow in the distal pulmonary circulation and eventually reduce left ventricular preload, which may lead to the loss of cardiac output (Figure 1). If the surgical team is involved at this point for the SPE or for institution of VA-ECMO, then haemodynamic instability could be avoided. Poor haemodynamics are independent predictors of the high 30-day mortality, and early SPE approach can improve operative survival up to 93%^[7]^. The right ventricle (RV) is thinner compared to the left ventricle, and that is the reason why RV is more sensitive to the acute afterload changes, and SPE is the swiftest modality to start “reverse remodeling of the RV dysfunction” by removing distal obstruction (Figure 2). Cardiopulmonary bypass (CPB) supports empty RVs by reducing preload and improves haemodynamic condition, giving opportunity to retrieve clots. A surge of attention in this field led to meaningful publications and unveiled continuous improvement in the SPE outcomes^[8,9]^. A large meta-analysis has reported overall in-hospital mortality between 16% and 24%, but single-centre retrospective studies are reporting single-digit mortality, and these diferences in the overall outcomes are because of individual hospital protocols and timing of the surgery^[10]^. Most of the publications reported that surgery was ofered in 35% of the cases after CPR, which is a known independent risk factor for poor outcomes. Results are better in the units where SPE is performed regularly and elected as a semi-urgent procedure before haemodynamic instability sets in. Our unit have published results of 82 cases with preoperative cardiac arrest in 14.64%, which was lower compared to many other reports (33.9%), and the reason behind these better outcomes can be contributed to our early operating policy^[6]^. Kalra et al.^[10]^ have reported an overall hospital mortality rate of 26.3%, while in our study, it was 8.54%. But even in our study, once the patient required preoperative CPR, then mortality rate escalated to 58.34%, and again it reinforces the fact about early intervention^[6]^. Five survivors in our study had cardiopulmonary arrest in the operating room, and CPR was performed prior to the sternotomy. Other seven patients went in cardiopulmonary arrest outside of operating room and could not be saved. Another unique complication seen among patients who required preoperative CPR was massive pulmonary haemorrhage through endotracheal tube (3/12 patients). Potential aetiology might be the pulmonary infarction caused by dislodgement of the clots from main pulmonary arteries into the distal circulation during cardiac massage (Figure 3). Other groups have also reported significant pulmonary haemorrhage caused by the pulmonary artery injury during clot removal from the branch arteries^[9]^.

**B.** Management of out-of-hospital cardiac arrest (OOHCA) patients with MPE is another debatable field, where there are no perfect answers. Multiple factors play important role in the decision making and outcome, like duration of cardiopulmonary arrest, effectiveness of CPR, return of spontaneous cardiac activity, associated primary pathology, and the time before reaching to the Emergency Department^[3]^. Early risk stratification is the key for good outcomes and, therefore, it is important to have quick tests, like serum lactate level, to triage high-risk cases and intervene early^[6,11]^. Our protocol is to insert arterial pressure line during CPR to assess cardiac output and effectiveness of the cardiac compressions. Patients presented with prolonged, unwitnessed, and ineffective resuscitation are ruled out for any surgical intervention and managed with systemic thrombolysis only. On contrary to previous reports, recent literature is suggesting improvement in the survival with the use of systemic thrombolysis during the resuscitation, and these findings further escalate the complexity in the decision making^[12]^.

**C.** Although the results of VA-ECMO in MPE with cardiogenic shock are encouraging, overall outcomes of VA-ECMO during CPR are still poor, with reported in-hospital mortality of around 75%^[4,13]^. Around 4-5% of the patients with MPE also have clots in the right atrium, RV (clots in transit), and acutely create instability by obliterating inflow and outflow valve. During ongoing CPR, establishing prompt CPB or VA-ECMO flows are paramount, but these intracardiac clots might get sucked in the venous cannula and cause distension of the heart by poor venous drainage. Various groups are recognising the importance of early haemodynamic stabilization by instituting VA-ECMO and managing patients either with thrombolysis or emergency SPE^[14]^. VA-ECMO insertion gives time for the patient transfer to the operating rooms with sustaining sufficient cardiac output. But “only VA-ECMO” support group has 2-3-fold higher mortality rate compared to the patients in whom VA-ECMO was followed by SPE^[4,15]^.

**D.** Systemic thrombolysis and catheter-based therapies (CBT) continue to be the class I indication in the management of MPE, while SPE is ofered in selected unstable cases, but thorough review of the contemporary literature gives interesting insight on this subject^[16]^. A recently published systemic review of 1,650 patients, who either underwent CBT (1,650 patients) or SPE (1,101 patients), presented similar in-hospital mortality if CBT or SPE was performed before cardiopulmonary arrest, but the advantages of SPE was complete clearance of the clots and more defnitive treatment in the long-term follow-up^[17]^. Comparing outcomes among these treatment modalities are not straightforward as SPE cases are sicker (21.4% had prior CPR) and have higher clot burden compared to CBT patients. Keeling et al.^[18]^ have reported a multicentre series and again reinforced that if SPE is performed timely, then good early outcomes can be achieved with low in-hospital mortality, but if the patient is having ongoing CPR, then mortality rate is 32.1%. Lee et al. have reported that overall use of thrombolysis and SPE in the management of pulmonary embolism (PE) is around 1% and 0.4%, respectively, and both modalities give similar early outcomes^[19]^. They have reported that patients in the SPE group had lower associated risks of early stroke, reintervention, and late recurrent PE compared with those in the thrombolysis group, and they have advocated SPE as it reduces future recurrence of PE.

**E.** It is mandatory to make notes of the duration of the haemodynamic instability with serum markers’ levels (D-dimers, troponins, and lactate), failure of the systemic thrombolysis, and duration of CPR before deciding the treatment modality. These are extremely relevant variables to decide whether to use “minimalistic approach” (VA-ECMO) or to perform index SPE surgery, with or without VA-ECMO support. Duration of the CPR makes big impact on the outcomes and that is the reason why in patients with OOHCA or in-hospital cardiac arrest where downtime is prolonged (30 minutes), surgeons have to critically analyse the situation and take consensual decision involving different treating teams^[20]^. George et al.^[11]^ have reported in their retrospective analysis that three groups where ECMO consistently gives poor outcomes are patients with malignancy, cardiac arrest prior to initiation of ECMO, and patients with serum lactate > 6 mmol/l. Although there are no randomized trials to support, ECMO-facilitated resuscitation has been increasingly used to assist early return of perfusion and support further resuscitation in order to mitigate the multi-organ dysfunction. But ECMO-assisted CPR should be used judiciously and most often attempted in cases with potentially reversible clinical conditions with least comorbidities.

## LEARNING POINTS

All available evidence supports prompt “risk stratification” and triage for the defnite treatment in patients with MPE. But, with the paucity of large randomized clinical trials, management of MPE with cardiopulmonary arrest is still an open debate with various choices. SPE can be a good option in these unstable patients, with centrally located massive clot with right ventricular strain and dilatation on echocardiography. Surgical outcomes are very poor in patients with OOHCA and in-hospital cardiac arrest (outside of operating room) and should be deferred in the favour of systemic thrombolysis with or without VA-ECMO support.

## References

[1] Iaccarino A, Frati G, Schirone L, Saade W, Iovine E, D'Abramo M (2018). Surgical embolectomy for acute massive pulmonary embolism: state
of the art. J Thorac Dis.

[2] Konstantinov IE, Saxena P, Koniuszko MD, Alvarez J, Newman MA (2007). Acute massive pulmonary embolism with cardiopulmonary
resuscitation: management and results. Tex Heart Inst J.

[3] Takahashi H, Okada K, Matsumori M, Kano H, Kitagawa A, Okita Y (2012). Aggressive surgical treatment of acute pulmonary embolism with
circulatory collapse. Ann Thorac Surg.

[4] Goldhaber SZ (2020). ECMO and surgical embolectomy: two potent tools to manage
high-risk pulmonary embolism. J Am Coll Cardiol.

[5] Edelman JJ, Okiwelu N, Anvardeen K, Joshi P, Murphy B, Sanders LH (2016). Surgical pulmonary embolectomy: experience in a series of 37
consecutive cases. Heart Lung Circ.

[6] Rathore KS, Weightman W, Passage J, Joshi P, Sanders L, Newman M (2020). Risk stratification using serum lactate in patients undergoing
surgical pulmonary embolectomy. J Card Surg.

[7] Crestanello JA (2018). Is it time to expand the indications for pulmonary
embolectomy?. J Thorac Cardiovasc Surg.

[8] Choi JH, O'Malley TJ, Maynes EJ, Weber MP, D'Antonio ND, Mellado M (2020). Surgical pulmonary embolectomy outcomes for acute pulmonary
embolism. Ann Thorac Surg.

[9] QiMin W, LiangWan C, DaoZhong C, HanFan Q, ZhongYao H, XiaoFu D (2020). Clinical outcomes of acute pulmonary embolectomy as the
first-line treatment for massive and submassive pulmonary embolism: a
single-centre study in China. J Cardiothorac Surg.

[10] Kalra R, Bajaj NS, Arora P, Arora G, Crosland WA, McGifn DC (2017). Surgical embolectomy for acute pulmonary embolism: systematic
review and comprehensive meta-analyses. Ann Thorac Surg.

[11] George B, Parazino M, Omar HR, Davis G, Guglin M, Gurley J (2018). A retrospective comparison of survivors and non-survivors of
massive pulmonary embolism receiving veno-arterial extracorporeal membrane
oxygenation support. Resuscitation.

[12] Bougouin W, Marijon E, Planquette B, Karam N, Dumas F, Celermajer DS (2017). Pulmonary embolism related sudden cardiac arrest admitted alive
at hospital: management and outcomes. Resuscitation.

[13] Meneveau N, Guillon B, Planquette B, Piton G, Kimmoun A, Gaide-Chevronnay L (2018). Outcomes after extracorporeal membrane oxygenation for the
treatment of high-risk pulmonary embolism: a multicentre series of 52
cases. Eur Heart J.

[14] Sharma V, Goldberg HD, Zubkus D, Shears LL, Kaczorowski DJ (2016). Successful management of cardiac arrest due to pulmonary embolus
using extracorporeal membrane oxygenation and ultrasound-accelerated
catheter-directed thrombolysis. Ann Thorac Surg.

[15] Goldberg JB, Spevack DM, Ahsan S, Rochlani Y, Ohira S, Spencer P (2021). Comparison of surgical embolectomy and veno-arterial
extracorporeal membrane oxygenation for massive pulmonary
embolism. Semin Thorac Cardiovasc Surg.

[16] Konstantinides SV, Meyer G, Becattini C, Bueno H, Geersing GJ, Harjola VP (2020). 2019 ESC guidelines for the diagnosis and management of acute
pulmonary embolism developed in collaboration with the European respiratory
society (ERS). Eur Heart J.

[17] Loyalka P, Ansari MZ, Cheema FH, Miller CC 3rd, Rajagopal S, Rajagopal K (2018). Surgical pulmonary embolectomy and catheter-based therapies for
acute pulmonary embolism: a contemporary systematic review. J Thorac Cardiovasc Surg.

[18] Keeling WB, Sundt T, Leacche M, Okita Y, Binongo J, Lasajanak Y (2016). Outcomes after surgical pulmonary embolectomy for acute pulmonary
embolus: a multi-institutional study. Ann Thorac Surg.

[19] Lee T, Itagaki S, Chiang YP, Egorova NN, Adams DH, Chikwe J (2018). Survival and recurrence after acute pulmonary embolism treated
with pulmonary embolectomy or thrombolysis in New York State, 1999 to
2013. J Thorac Cardiovasc Surg.

[20] Laher AE, Richards G (2018). Cardiac arrest due to pulmonary embolism. Indian Heart J.

